# News and Miscellany

**Published:** 1882-04

**Authors:** 


### NEWS AND MISCELLANY.

HONORS TO A VETERINARY SURGEON.—Mr. H. Bouley, member of the Institute, Paris, Inspector General of the Veterinary Schools, France, has been raised to the rank of Commander of the Legion of Honor.—*American Veterinary Review.*

NEW VETERINARY SCHOOL—Was opened at Lemberg, Poland, under the direction of Dr. Seifmann, of the Kazan Veterinary School, with Drs. Baranski and Kaydi as professors.

WEIGHT OF A COW'S BRAIN.—The weight of the brain of the cow at the third year of life is inferior to that of man. The average weight of the adult human brain is 48 oz. and that of the cow about 16 oz. The weight of the brain of a child three years old greatly exceeds that of a cow of the same age.

THE CELEBRATED HUNTING DOG, LINCOLN, owned by Harry Bishop, of Tennessee, which has been at New Albany for treatment on account of a railroad accident, died last week. The dog cost $2,950 in England.

GREENWOOD CEMETERY REPORT.—The successful introduction of the grey squirrel is referred to as having added a new and attractive feature to the rural character of the cemetery. "Forty of these graceful creatures," says the report, "were there set free early in the Spring, and at once made their homes in the boxes provided for them in the trees. Such is their rapid increase that, before the close of the year, they numbered in all about 150." The destruction of objectionable animals and vermin in the cemetery has been "continued with diligence." The result of this warfare was the extinction of a fox, the second one ever killed in the cemetery, 11 moles, 25 rats, 70 dogs, 199 cats, 75 snakes, 536 ground mice, and 3,051 chipmunks. The trustees point out the fact that not a skunk or muskrat was found during the year.

UNDER PERFECT CONTROL.—To show how completely Captain P. F. Alba has subdued and mastered, by the law of intelligent kindness, that horse once known as the man-eater, we will state that on last Monday afternoon, while the Alabama State Artillery were firing a salute on the wharf in honor of the arrival of the Emperor Felix, Captain Alba rode this horse down between the two guns and sat him there while the firing was going on. Sometimes both horse and rider were invisible from the smoke of the rapid discharges. The horse seemed to have no fear, but rather to enjoy the excitement.

SPANISH CATTLE.—In consequence of the recent arrival of a cargo of Spanish cattle at Jersey, many of which were suffering from disease, the Veterinary Department of the Board of Trade has informed the States Assembly of that Island that, unless stringent measures be adopted in respect of such cattle, it may be found necessary to order the slaughtering of Jersey cattle landed in England for exhibition or otherwise.—*English Live Stock Journal.*

VIVISECTION AGAIN.—Two prizes, one of 2,000f. and another of 1,000f., have been offered by the Danish Society for the Protection of Animals for the best and second best essays showing how far recently killed animals may be substituted for living ones in pursuing physiological experiments. The essays, which may be written in Danish, Swedish, English, French, or German, must be forwarded to Mr. A. de Haxthausen, the President of the Society, at Copenhagen, before September 1st of the present year. The object is to abate those investigations in which "animals are subjected to great and lingering agony."

BEARS OF BERNE.—Berne, the venerable capital of the Helvetic Confederation, has recently been stricken by a grievous bereavement. On Christmas eve, Mani, the elder of the two tutelary bears, dear to every Bernese heart, which have for so many years past enjoyed honorable publicity in the Fosse aux Ours of the Ursine City, took his departure for that bourne from which no bear, however estimable and popular, has ever yet been known to return. But for the fact that Mani, some twenty-one years ago, did to death an Englishman who imprudently tumbled into the pit inhabited by the Bernese Bruin—at that time in the heyday of impetuous and inconsiderate youth—we should share the regrets of his mourning fellow-citizens to a greater extent than the remembrance of that ill-judged act will at present permit us to do. As it is, we can but offer them the expression of a mitigated condolence, and of the hope that Mani's successor, should a stray Briton fall into his clutches, will prove the quality of mercy not to be quite so lightly strained in his case as it was in that of the too-choleric departed.

NEW HORSE DISEASE IN PHILADELPHIA.—Within the last few days stablemen throughout the city have been surprised at the appearance of a peculiar disease among many of the horses under their charge, affecting the feet and ankles of the animals in a most unusual way. The first symptoms are fever and inflammation upon the parts around the pasterns and below the fetlock. The following morning there are running discharges of thick yellowish matter, and afterward pus begins to flow freely. The horse becomes lame and dispirited and is totally unfit for work. Experienced horsemen appear to be at a loss to comprehend fully the nature of the disease, but all are united in the opinion that it has been caused by the salted railroad tracks. The treatment to be followed, according to veterinary surgeons, is to poultice the ankle with bran or flaxseed to remove the fever, and then to use astringents. The horses must have perfect rest until all evidence of the disease is gone, for exercise will only tend to reopen the sores. A few of the street railway horses are afflicted, but the drivers are reticent in speaking of the matter.

CLYDESDALE HORSES.—Over eighty head of Clydesdale horses were shipped last week by the Glasgow Clydesdale Horse Breeding and Exporting Company to Colonel Robert Holloway, of Alexis, Ill. Says the London *Chambers of Agriculture Journal*, of Feb. 20: "When it was intimated last July that a company had been formed for the purpose of exporting Clydesdale stock to America, a number of persons thought the scheme impracticable. Two large shipments left Scotland last Autumn, and the movement thus organized promises before long to become an established trade. The vast prairies of the West offer magnificent opportunities for breeding horses, and the rapid growth of the cities in the States has created a demand for heavy draught animals hitherto unprecedented."

SCARLET FEVER AMONG HORSES.—The so-called "pink-eye" of horses is attended with soreness of the throat, difficulty of swallowing, swelling of the glands of the neck, dropsy of the legs and disorder of the kidneys; the affection of the eye is the least part of the trouble. This disorder the best French veterinary surgeons have pronounced to be scarlet fever, with or without the addition of diphtheria. There are 10,000 horse stables in the city, housing 85,000 horses, of which about 6,000 die each year, some of them of pink-eye. Many of these stables are in the best parts of the city; the majority of them are dirty and unsavory, and in few or none are disinfectants used. Gypsum is perhaps the best absorbent and deodorizer, although pine-wood shavings and saw-dust are very good. Cerebro-spinal meningitis is also common among horses. It seems to be an acute typhoid, or septic rheumatic inflammation of the membranes of the brain and spinal cord, produced by exposure to cold and wet and impure air. It is not directly contagious, but is conveyed in the same way, perhaps, that typhoid fever is.—*Times.*

How Street-Car Horses are Fed.—The ration for street-car horses in this country is now pretty definitely fixed in all our cities. For summer this ration is half oats and h lf corn, ground together, sixteen pounds to each horse, with twelve pounds of cut hay. In winter, sixteen pounds of corn meal, with the same amount of hay, forms the ration. Corn meal alone in summer is too heating, but in winter the corn meal seems well adapted to keep up animal heat and condition, and, being cheaper than oats, is generally adopted in New York city; but in many other cities half oats is used the year round. If these companies would substitute clover hay for timothy, corn meal would make a well-balanced ration. The clover would make up for the deficiency of the corn meal in muscle-sustaining food. Clover is rejected because it is liable to contain dust, which may develop heaves; but this fear is groundless under the plan now adopted of moistening the cut hay and mixing the meal with it. It is fed in a damp condition, and, therefore, no dust can be present to affect the lungs. Clover hay is not properly appreciated as a food for horses. It has a higher value than timothy, and is usually sold at $2 to $3 per ton lower in market. Corn is too much used by some car c mpanies. It is a fattening and "heating" food *par excellence*, and not a muscle builder.—*National Live Stock Journal.*

Cattle Troughs and Infectious Diseases.—The cattle troughs that have been placed in so many parts of London have not been an unmixed blessing to the animals for whose benefit they were instituted, and there is too much reason to fear that infectious diseases have been largely circulated among the stables of the metropolitan district by means of these troughs. The subject was referred to at the last meeting of the St. George's, Hanover Square, Committee of Works, when a letter was read from the Metropolitan Drinking Fountain and Cattle Trough Association, Westminster, requesting permission to place a cattle trough in the King's Road, Euston Square. This proposal met with opposition, one of the members of the committee moving that the permission requested be not given, on the ground that these troughs were a cause of disease being spread among horses. In the end, the committee decided to state, in reply to the application, that in their opinion the troughs, as at present constructed, are sometimes the means of spreading disease, owing to the circumstance that there is no stream of water through them. The improvement suggested might be made, we imagine, without much difficulty, and would remove the principal objection to the troughs.—*St. James' Gazette.*

Birth of an Elephant in London.—If the prognostications of the naturalists be correct, there will shortly be born a real English elephant in the Zoological Gardens. The first of its race who has entered Europe, other than an alien. since prehistoric times, it will form a notable addition to Western curiosities. Time was when the mammoth and the elephant no doubt looked upon England and the rest of Europe as their happy hunting-ground, and roamed at their own sweet will where they listed. But London in those primitive days was not yet dreamed of, and the Zoological Gardens extended all over the land. Since then elephants have not bred in Europe, and have shown signs of disappearing even from Asia; while the constant demand for ivory in Africa must seriously have diminished the number of tusks in the "dark continent." For an elephant, then, to be born in England is an event of no small interest, and a direct proof of the strides which the scientific knowledge and treatment of animals have attained. As a British elephant, the newcomer will be absolutely the only one that existed, for when the foot of the tropical fauna trod this part of the globe before, there was no Great Britain existing, and what are now islands were joined hard and fast to the main land. It is certainly against the probabilities that, after the lapse of so many ages as have passed since then, the elephant should once more appear as a native of the North.

Hints for Curing Balky and Unruly Horses. — 1. Place yourself against his near shoulder, and with your right hand seize the foreleg just above the pastern joint, raise the foot as high as you can, and force it forward. This will in most instances prove effectual.

2. Stand in front of the horse and draw his ears through your hands for two or three minutes, stroke his nose, and lastly take him by the head, and in an encouraging voice urge him to go forward.

3. If in single harness, and with a two-wheeled vehicle, take your animal's head and turn him round four or five times, keeping the inside wheel as near as possible in the same place.

4. Tire your steed out by remaining perfectly quiet until he starts of himself. I once sat in my cart nearly two and a half hours in this way.

Now and then a horse is met with that refuses to draw at all. Put him in a cart in a shed, and keep him there till he walks out. In one instance that came to my knowledge, the obstinate one was thirty-six hours in the shafts before he gave in.

If it is intended to cure a restive horse, he must be used solely by one and the same person, and caught young; and let his rider or driver bear in mind that with both restive and nervous horses the voice will prove more effectual than the whip. Stick to your nag if possible, under all circumstances; for, rely upon it, if he can get away from you, he will redouble his efforts to do so again. Unfortunately, there is always a risk in buying a once willful steed, for in fresh hands he may revert to his old tricks.—*London Live Stock Journal.*

The Goat Society.—The Duke of Wellington, K.G., President of the British Goat Society, occupied the chair at the annual meeting of members, held on Monday last. There was a good attendance. The Hon. Sec., Mr. H. S. Holmes Pegler, read the report. The number of members now is 242. A stud-goat register has been established. The system of supplying goats to cottagers works well. The large demand for goats and the increasing use of goat's milk as a food for infants have led the committee to consider the desirability of starting a limited company for the importation, breeding and supply of goats. The report having been adopted,

Gen. Burnaby, M.P., spoke strongly in favor of goats' milk. Every village had its invalids and its young folk to bring up, and it was of the greatest importance that they should be able to obtain so health-promoting an article of diet. Not only was the goat one of the nicest and healthiest animals to have in the stable, but if it were wished to cure a kicking horse, this purpose could be easily effected by introducing a goat into the stable.

The President said it might be taken for granted that milk from the goat was very much better than that yielded by the cow, and it was very desirable that means should be taken to overcome the prejudice which existed against goats' milk.

The meeting unanimously approved the proposal to form a Goat Supply Company. It was also resolved to form a deputation to the Privy Council, with a view to obtain the removal of the restrictions now laid upon the importation of goats from abroad, and to urge the advantage of having a port on the east coast for quarantine.

Many noblemen and ladies, as well as those of other rank in England, belong to the Goat Society, and the members and interest in the society are constantly increasing. Kid meat is found as delicate as the finest of lamb; and the milk of the ewes is richer than that of the Channel Islands cows, and what is more important to add, being easier of digestion, it is better for children and invalids. These goats are selected and bred for superior milking qualities, and many a poor family can keep them when unable to support a cow. It is for such, more particularly, that English landlords are promoting the breeding of goats. There are millions of acres of mountainous land in the United States too broken in surface and rocky, or of too poor a soil to support cattle, and yet they are admirable for the support of goats; and the day will come when thousands of these will undoubtedly be found pasturing on these lands, greatly to the profit of their owners.—*National Live Stock Journal.*

Ostrich Farming.—The American Consul at Buenos Ayres renews the suggestion that ostrich farming be undertaken in this country. The destruction of the birds has progressed until the species has become entirely extinct in Arabia, and nearly so in South Africa, while in the Argentine Republic it is now to be found in any considerable numbers only on the extreme western and southern frontiers. The only hope of preserving the bird from becoming extinct like the dodo is in domesticating and propagating it on ostrich farms, as is already done successfully at Cape Town, and has been lately attempted in the Argentine Republic. A range of 6,000 acres, in an open state, will readily subsist and keep 5,000 birds, and this estimate is regarded as too severe; with suitable inclosures the birds cost little to keep, as they thrive on ordinary grass pasturage, although a grain allowance improves the plumage; almost any soil answers, although one containing gravel or small stones is preferable; the birds seldom sicken, graze during the day, require little watering, and lie down to sleep at night wherever they happen to be; they "drive" very readily, need no regular herdsman when properly fenced, breed at four years for the female and five for the male; one pair will hatch out a brood of ten to fifteen four times a year; one egg a day is laid by the female, and when fifteen to twenty are accumulated she sits on them in the day, the male relieving her in the night; forty-two days are required, and if the chicks are removed after hatching, the female at once recommences laying—in this respect being very unlike the common hen. Chicks a month old are worth $50 at Cape Colony. At six to eight months they are plucked, and at the like interval progressively; mature birds produce twenty-five long white feathers from each wing, worth $5 a piece at Cape Town; chick feathers are worth only $5 per bird, but the next pluckings yield $40 to $150 per bird, and at an assumed average of $75, there will be a gross income of $150 in sixteen months, or about $112 per year per bird; with young and growing birds the least hopeful view must assume at least seventy-five per cent. annual profit from feathers, with also a doubling in value of the birds; the gross income from the chicks produced by one pair is from $2,000 to $3,000 per year. These figures are from the pamphlet of a firm in Buenos Ayres who are importing birds from Cape Town for stocking farms, and must be received with some allowance accordingly; this firm had lately brought in a lot of 200, which came from Cape Town by sail, losing only eight. Prices for imported birds have ranged from $1,000 to $1,250 each for full grown. The bird is, however, long lived; some call it a centenarian, but the usual rule of calculation makes its term twenty-four to thirty years. The Consul says it is a popular error to suppose that the ostrich is adapted chiefly to the desert; probably it took to the desert to escape its many enemies, and experience has so well proved that it will thrive under extreme temperatures that it would evidently like succulent grasses much better than desert fare. At Cape Colony, ostrich farming has now been fourteen years established, and the Consul, who claims to have considerably investigated the subject, says the business ought to thrive, especially in the United States. The tempting figures presented suggest the possibility of overdoing feather production. As to this, he says that the feather exports from Cape Colony fourteen years ago were $350,000 in value, chiefly from wild birds. Now the value is $4,500,000; yet prices have not declined. All the female sex demand the feathers. Present profits are such as make ostrich farmers careless of the distant future; but fear of any collapse in the demand is almost absurd.

The Photographer of the Galloping Horse.—A lecture by Muybridge of Palo Alto at the Royal Institution, with the exhibition of instantaneous photographs illustrating the movements of animals, attracted a distinguished audience, including the Prince and Princess of Wales, the Duke of Edinburgh, Mr. Tennyson, and Mr. Alma-Tadema. His demonstrations of the inaccuracy of much of ancient and modern art, and the contrasts exhibited between conventional and real movements, especially the absurdity of Frith's horses in the picture of Derby Day, excited the keenest interest. The Prince of Wales repeatedly questioned the lecturer, thanking him afterward. The lecture was repeated at the Royal Academy, most of the leading artists being present. The result was that even animal painters were convinced of the novelty and importance of Muybridge's observations

More of the Cattle Plague.—The name of Augustus Denniston, signed yesterday to a proclamation for the enforcement of the law relative to infectious and contagious diseases in animals, drew attention to the fact that a successor to Gen. M. R. Patrick had been appointed. It appears that Gen. Patrick has not performed the duties of the office for some months, but has accepted and is performing the position of Governor of the National Soldiers' Home of Dayton, Ohio. Meanwhile his deputy, Alexander Orr Hopkins, has acted in his place. Since the law was passed in 1878, 838 infected cattle have been destroyed according to law, and their owners indemnified at an average rate of about $50 a head, which is calculated to be two-thirds the value of the animals. About 500 more cattle have been destroyed by their owners after it had been discovered that they had been exposed to infection, although the disease had not developed.

When the law was passed the "lung plague" was spreading rapidly. In Blissville, L. I., stables alone there were 240 infected cattle. By the application of vigorous quarantine the disease was almost stamped out. It has recently reappeared in more force, and it was this that induced Gov. Cornell to appoint Mr. Denniston on March 9th. It has been found that there is more or less of the disease in Brooklyn, in Westchester County, and in New York city stables. A herd of thirty-one infected cattle was recently exterminated in Putnam County. A prolific cause of spreading the disease is the habit of sending milch cows from one dairy to another on trial. It is intended to appoint inspectors to search out these cases.

Mr. Denniston was busy yesterday conferring with Superintendent Walling to secure the co-operation of the police, and visiting the Union Stock Yards and other cattle depots. The office will remain at 29 Fulton street, Brooklyn, for the present, and be open in business hours. The permits granted by Gen. Patrick have been revoked, and dealers must take out new ones. What are called "running permits" will be given to regular dealers to convey cattle from New York to Brooklyn, Staten Island, and New Jersey. But in special cases of bringing cattle into the city, separate permits are required. No fee is charged for a permit, for the expenses of the Cattle Commission are paid by the State.

The "lung plague" was known in the days of Aristotle, 380 years before Christ. Its peculiarity is that the lung is solidified by accumulations until, instead of weighing four pounds, the organ weighs over forty pounds. The breathing apparatus is thus made useless. The disease is infectious and contagious, and cattle dying of it are not fit for human food. The disease made its appearance in Holland in 1833, and it is estsmated that the damage by it, in England alone, has been $10,000,000. It first made its appearance in Brooklyn in 1843, in a cow bought by Peter Dunn from a shipmaster. This cow infected the cattle of the west end of Long Island, and thence the disease spread.

Mr. Denniston is from Orange County, where they know something about milch cows. He is a son of ex-State Comptroller Denniston.

BURIAL-GROUNDS FOR ANIMALS.—The proposal which seems to have been made to provide "a burial-place for pet animals, dogs, pussy-cats, and little birds," accessible to Londoners, is, as the *Pall Mall* has justly remarked, not so grotesque as it seems at first. We have known more than one case in which many miles have had to be traveled by the mistress or master of the most faithful companion either had ever had, in order to bury its poor little body at all. Most London houses have no gardens, and most people would shrink from casting the body of a creature of which they had been honestly fond on to the dust-heap, to be taken away by the scavenger. There is even more of the feeling that the body of a dog, a cat, or bird, is identified with the living creature than there is in the case of man, where the almost universal belief in immortality operates to excite a new train of associations in a totally different direction. Still, there are very few who identify the creatures of which they have been fond so completely with the mere bodies of them as to be willing to have them stuffed (as it was said that Jeremy Bentham was stuffed), to mock the memory with a dreadful parody on what they were. Hence, both for rich and poor, some burial-ground is necessary, unless the bodies of the dead favorites are to be spurned as mere garbage—a fate from which even the mildest degree of genuine sentiment necessarily saves them.—*London Spectator.*

FREE-MARTINS. — The twin-heifer born with a bull-calf is commonly called a free-martin, and, as a general rule, is barren. When twins are of the same sex, they breed as well as those not twins; and there are a good many cases of free-martins breeding regularly. But it seems that when a female and male calf are produced together, the female generative organs are generally imperfect, partaking of the character of both sexes. It is generally found that such twin-heifers take on a masculine form, looking more like steers than heifers. The free-martin usually feeds well. The twin bull-calf is a perfect male, and will be as prepotent as if not a twin.— *National Live Stock Journal.*

MONKEYS.—If the skeletons of an orang-outang and a chimpanzee be compared with that of a man, there will be found to be the most wonderful resemblance, together with a very marked diversity. Bone for bone, throughout the whole structure, will be found to agree throughout the general form, position and function, the only absolute differences being that the orang has nine wrist bones, whereas man and the chimpanzee have but eight; and the chimpanzee has thirteen pairs of ribs, whereas the orang, like man, has but twelve. With these two exceptions the differences are those of shape, proportion and direction only, though the resulting differences in the external form and motions are very considerable. The greatest of these are that the feet of the anthropoid or man-like apes, as well as those of all monkeys, are formed like hands, with large opposable thumbs fitted to grasp the branches of trees but unsuitable for erect walking, while the hands have weak small thumbs but very long and powerful fingers, forming a hook rather than a hand, adapted for climbing up trees and suspending the whole weight from horizontal branches. The almost complete identity of the skeleton, however, and the close similarity of the muscles and of all the external organs, have produced that striking and ludicrous resemblance to man which every one recognizes in these higher apes and, in a less degree, in the whole monkey tribe; the face and features, the motions, attitudes and gestures being often a strange caricature of humanity. Let us, then, examine a little more closely in what the resemblance consists, and how far, and to what extent, these animals really differ from us. Besides the face, which is often wonderfully human—although the absence of any protuberant nose gives it often a curiously infantile aspect—monkeys, and especially apes, resemble us more closely in the hand and arm. The hand has well-formed fingers and nails, and the skin of the palm is lined and furrowed like our own. The thumb is, however, smaller and weaker than our own, and is not so much used in taking hold of anything. The monkey's hand is, therefore, not so well adapted as that of man for a variety of purposes, and cannot be applied with such precision in holding small objects, while it is unsuitable for performing delicate operations, such as tying a knot or writing with a pen. A monkey does not take hold of his nuts with his forefinger and thumb as we do, but grasps it between the fingers and the palm in a clumsy way, just as a baby does before it has acquired the proper use of its hand. Two groups of monkeys—one in Africa and one in South America—have no thumbs on their hands, and yet they do not seem to be in any respect inferior to other kinds which have it. In most of the American monkeys the thumb bends in the same direction as the fingers, and in none is it so perfectly formed as our thumbs are. This shows that the hand of the monkey is, both structurally and functionally, a very different and very inferior organ to that of man, since it is not applied to similar purposes, nor is it capable of being so applied. When we look at the feet of monkeys we find a still greater difference, for these have much larger and more opposable thumbs, and are therefore more like our hands; and this is the case with all monkeys, so that even those which have no thumbs on their hands, or have them small and weak and parallel with the fingers, have always large and well-formed thumbs on their feet. It was on account of this peculiarity that the great French naturalist, Cuvier, named the whole group of monkeys Quadrumana, or four-handed animals, because, besides the two hands on their fore-limbs, they have also two hands in place of feet on their hind limbs. Modern naturalists have given up the use of this term, because they say that the hind extremities of all monkeys are really feet, only those feet are shaped like hands; but this is a point of anatomy, or rather of nomenclature, which we need not here discuss.—*Contemporary Review.*

